# Virucidal Efficacy of Laundering

**DOI:** 10.3390/pathogens11090993

**Published:** 2022-08-31

**Authors:** Nadine Merettig, Dirk P. Bockmühl

**Affiliations:** Faculty of Life Sciences, Rhine-Waal University of Applied Sciences, 47533 Kleve, Germany

**Keywords:** virus, laundry, detergent, oxygen bleach, disinfection

## Abstract

Viruses contribute significantly to the burden of infectious diseases worldwide. Although there are multiple infection routes associated with viruses, it is important to break the chain of infection and thus consider all possible transmission routes. Consequently, laundering can be a means to eliminate viruses from textiles, in clinical settings well as for domestic laundry procedures. Several factors influence the survival and inactivation of microorganisms, including viruses on hard surfaces and textiles. Therefore, textiles should be regarded as potential fomites. While in clinical and industrial settings laundry hygiene is ensured by standardized processes, temperatures of at least 60 °C and the use of oxidizing agents, domestic laundry is not well defined. Thus, the parameters affecting viral mitigation must be understood and prudently applied, especially in domestic laundering. Laundering can serve as a means to break the chain of infection for viral diseases by means of temperature, time, chemistry and mechanical action.

## 1. Introduction

When trying to control the spread of viral pathogens it is crucial to consider all means suitable for breaking the potential chain of infection. In this regard, the virucidal performance of domestic laundry processes might act as one important parameter to reduce virus transmission [1,2], although surfaces have been shown only to play a minor role in the transmission of respiratory viruses, such as SARS-CoV-2 [3,4]. The COVID-19 pandemic has certainly created a growing demand for an effective hygiene framework to deal with viral pathogens in everyday life settings. Although common domestic hygiene standards should always be applied in a targeted way [5], e.g., hand hygiene, respiratory hygiene and general home hygiene, hard surfaces and textiles can be regarded as fomites and thus need to be addressed to lower the infection risk by viral pathogens including enteric viruses (norovirus and rotavirus), respiratory viruses (influenza and coronavirus) and other viruses, e.g., herpes simplex virus and poliovirus [6]. The main building blocks of the viral structure are the nucleic acid core and the surrounding protein capsid for non-enveloped viruses and an additional lipid bilayer for enveloped viruses, harboring surface proteins or glycoproteins which mediate the connection to specific receptor sites on susceptible host cell surfaces. The structure of surface proteins is determined by the viral nucleic acid, while the lipid bilayer is derived from the host cell membrane. Within the classification of enveloped and non-enveloped viruses, their physico-chemical properties can be further distinguished according to their lipophilic and hydrophilic nature [7]. When assessing the environmental stability and the efficacy of virucidal means, these properties are considered crucial.

## 2. Viral Persistence and Susceptibility to Biocidal Agents

When it comes to viral persistency during the laundry process is it important to differentiate between enveloped and non-enveloped viruses due to the inherent differences in virion stability, which is influenced by several factors. Apart from the presence of an envelope, the type of nucleic acid and the general sensitivity of viral proteins to pH changes is important [8]. Other environmental parameters like temperature, humidity, sunlight and the presence of organic matter can influence viral persistence as well [9,10]. In particular, temperature greatly impacts the survival and stability of viruses. While some viruses can be inactivated within minutes at high temperatures (>50 °C), other, non-enveloped viruses can remain infectious for days or months at ambient temperatures [2,8,11]. The persistence of enveloped viruses on surfaces also depends on the material surface porosity. In general they survive longer on plastic and stainless steel than on porous surfaces (i.e., paper) as droplets of water may form on hydrophobic surfaces which can harbour viral particles [12]; it can be assumed that mainly electrostatic forces are mediating the interaction between hydrophobic groups on the virus and the surface [13]. Thus, viruses such as SARS-CoV-2 generally have a high ability to contaminate a wide range of porous and non-porous surfaces [13,14]. Besides a direct spread via droplet or aerosols, viruses may also be transferred via surfaces as hard surfaces. Likewise, textiles might serve as a viral reservoir [15,16] and should be considered to be horizontal transmission pathways [17,18], especially, since some viruses such as influenza viruses and coronaviruses (CoV) have a high potential to survive on dry surfaces [18]. For instance, CoV have been shown to persist for up to nine days on hard surfaces [10,16,19] and up to 48 h on textile fabrics [20]. What limits the infection from fabrics and/or other surfaces is that transfer rate of the virus from these surfaces is low which mediates some of the risks. Detergents affect the lipid bilayer membrane of the virus envelope by disrupting the hydrophobic interaction between the nonpolar fatty acid chains and can render viruses inactive. The susceptibility of viruses to detergents and disinfectants is related to their lipophilic or hydrophilic character, the general efficacy of detergents and disinfectants against viruses depend on the presence of a viral envelope and the lipophilicity of the virion (Table 1) [21].

The efficacy of chemical substances against viruses is mainly tested by comparing the amount of virus particles that are able to infect host cells in a cell culture model with or without antiviral treatment. The experimental conditions are usually defined to address in normative methods, such as EN 14476 or EN 16777 [24,25]. Virucidal efficacy test methods use surrogate viruses as test strains representing a group of relevant viruses. According to EN standards, substances effective against enveloped viruses only are often referred to as having a limited virucidal activity, whereas for a full virucidal activity claim an efficacy against enveloped and non-enveloped viruses has to be proven (Table 2) [2,26,27,28]. As mentioned above, non-enveloped viruses are generally more resistant to chemical agents such as detergents or even biocides than non-enveloped viruses and bacteria [2,29]. For enveloped viruses, cleaning procedures using common detergents often lead to considerable reduction factors [2]. Thus, even products that have not undergone normative virucidal efficacy testing may exert a good virucidal activity, especially on non-enveloped viruses. For example, Chin et al. demonstrated the antiviral effect of plain soap solution against SARS-CoV-2 [19]. Different biocidal agents have been proven to be active against SARS-CoV-2 in suspension tests, e.g., ethanol, 2-/1-propanol/, glutardialdehyde, formaldehyde, povidone iodine, benzalkonium chloride, sodium hypochlorite and hydrogen peroxide [10,16], although their efficacy strongly depend on several parameters and not all of them are used in laundering. Typical reduction factors (log_10_) range between ≥3 and ≥5 [16].

## 3. Factors Influencing the Antiviral Efficacy of Laundering

Like the cleaning performance, the antimicrobial effect of laundering follows the principle introduced by Herbert Sinner (1960) according to which a washing process is determined by four variables: temperature, mechanical action, chemistry and time [31].

### 3.1. Temperature

Temperature directly affects the inactivation of microbial cells and viral particles on reduction on laundry items by thermal impact; it also helps in physico-chemical removal of organic matter and plays a role in the activation of peracids. Studies suggest that temperatures of 60 °C might be able to completely reduce the vast majority range of microorganisms on textiles, including non-enveloped viruses, even without the use of activated oxygen bleach [11,32,33,34,35,36,37,38,39,40,41,42]. For decades, a trend towards lower temperatures has been observed, which comprises the major means to save energy during the washing process and to promote clothes longevity; however, this also has been shown to decrease the antimicrobial performance of laundering; moreover, it has been shown that particularly enveloped viruses are readily inactivated even when lower temperatures and a bleach-free detergent is used [2]. In North America laundering conditions typically are much lower than European with the cold wash being on average 16 °C compared 30 °C for Europe with minimal impact on overall health. Thus, laundering at lower temperatures can considered to deliver a sufficient hygiene level, unless non-enveloped viruses are concerned.

### 3.2. Chemistry

Even without the use of antimicrobial agents, the detergency effect will lead to a considerable decrease of the microbial cells on a fabric surface, which can be attributed to the mechanical removal rather than a killing effect; however, adding commonly used biocidal substances such as bleaching agents or quaternary ammonium compounds might increase the antimicrobial efficacy [2,43,44,45,46,47,48,49]. Gerba et al. reported the effect of washing and drying in a home washing machine on enteric viruses (Adenovirus, Rotavirus, and Hepatitis A virus) [48]. The enteric viruses remained infectious throughout laundering without bleach and transmission from contaminated to uncontaminated swatches occurred, while a great impact on 99.99% virus reduction by bleach was observed [48]; furthermore coronaviruses are effected by bleach (concentration 0.1%), either Ethanol (62–71%) showed to be quite effective (>4 log) [10]. Especially SARS-CoV-1 und SARS-CoV-2 are described as unusually stable beside the envelope, even though they are rather lipophilic and sensitive to solvents and surfactants [21]; moreover, a wide pH stability could be shown for SARS-CoV-2 [21]. The virucidal efficiency of current laundering processes for enveloped viruses is due to the virion sensitivity to detergents and aided by bleach and they can be inactivated even at 20–30 °C [2,50]. Virus particles, enveloped or not are able to survive washing at 30 °C without detergent and can contribute for transmission, so that recommendations for the inactivation, use detergents or elevated temperatures [2,11,21,50]. Traditionally, in many parts of the world, chlorine bleach has been used for this purpose, whereas in other countries activated oxygen bleach (AOB) can be predominately found. The active ingredient percarbonate releases hydrogen peroxide in aqueous solutions at higher temperatures. Using bleach activators such as TAED (tetraacetylethylenediamine) or NOBS (sodium nonanoyloxybenzenesulfonate) can push this reaction below 60 °C and can thus significantly increase the antimicrobial efficacy even at lower temperatures [34,38,41]. The use of quaternary ammonium compounds (QAC) like benzalkonium chloride (BAC) and dimethyl didecyl ammonium chloride (DDAC) is common for hygiene rinse aids since anionic surfactants which are widely used in laundry detergents are not compatible with those. QACs can interact with the surface of negatively charged textiles, and thus providing a persisting antimicrobial effect. The exerted antimicrobial efficacy highly depends on the type of microorganism and residual detergent left in the rinse. While the use of QACs results in a high reduction of gram-positive bacteria even at low concentrations, fungi or Pseudomonads are much less affected by these biocides [51]. There is no comprehensive data on the antiviral efficacy of QACs in laundering, although these substances are active against certain viruses as well [52]. Chin et al. described the viral reduction below levels of detection, when 0.10% of benzalkonium chloride is used against SARS-CoV-2 [19].

### 3.3. Time

As proposed in the Sinner’s principle, it has been shown that a decrease in temperature can be compensated by increasing other variables, in particular the wash cycle time [34]; however longer washing cycles cannot completely restore the antimicrobial performance of laundering for certain microorganisms at very low temperatures [34]; it must be noted that this has been proven using bacterial and fungal test strains, but has not applied to virucidal effects so far. Nevertheless, it can be assumed that at least a limited compensation of lower temperatures by longer times will also be seen for virucidal effects. Honisch et al. described the interplay between time and temperature in current washing machines that aim to decrease the washing temperatures in order to save energy and in turn exhibit very long programme durations [34,53].

### 3.4. Mechanical Action

Finally, the construction type of the washing machine (i.e., horizontal vs. vertical axis) can be regarded a factor influencing the antimicrobial efficacy by mechanical action. Although evidence is still poor, it was shown by Honisch et al., that the mechanical action of the washing machine might help to physically remove microbial cells from textiles [44]. At least for enveloped viruses, carrying a cell membrane as the outer layer, these principles may apply as well.

## 4. Studies on the Antiviral Efficacy of Laundering

In terms of their role in the transmission of infectious diseases, textiles and laundering have not been studied intensively so far. Bloomfield et al. (2011) compiled available studies on the potential infection risk associated with contaminated textiles, also regarding viruses, resuming that textiles are considered potential fomites, also for viral diseases [15,48]; however, due to the low frequency of association it may not be a large source of transmission. Some studies described the microbial community associated with laundry as a resulting consortia from skin-associated bacteria, microorganisms from the environment and from washing machine biofilms [54,55,56,57]. As mentioned before, laundering can be used to disinfect contaminated textiles; however, when addressing a laundry-related risk of virus infections, not only the reduction by the laundering process itself has to be considered. Likewise, the specific interaction between virus and textile and the viral survival vary significantly depending on the virus species and the way of handling textiles in the laundry process [53]. Parameters like ambient organic matter or the ability to associate with surfaces via connection by the spike glycoproteins as anchors, stabilize the virion [49]. Enteric viruses are far more resistant to laundering procedures and antimicrobial agents. In general, non-enveloped viruses are more difficult to inactivate, whereas enveloped viruses are easier to inactivate in a washing process, because the phospholipid envelope can be disrupted by the detergent. Some non-enveloped enteric viruses have been proven to survive longer on textiles, and Poliovirus survives at room temperature on cotton up to 84 days [58]. Higher temperatures (30–40 °C) decreases the duration of persistence of coronaviruses, i.e., MERS-CoV, Alphacoronavirus 1 (TGEV), MHV, while lower temperature (4 °C) increase the persistence of MHV and Alphacoronavirus 1 (TGEV) up to 28 days [10]. HCoV 229E seems to be stable at room temperature and 50% relative humidity (RH) [10], whereas temperature at 6 °C seems to have a greater impact on enhanced survival rates than RH [59]. In general the influence of humidity on persistence has been described inconsistently [60].

Scientific studies on laundry hygiene are mostly focused on bacteria and fungi, whereas viral pathogens have been considered much less [53,61]; however, there are some studies dealing with viruses on textiles in particular and the antiviral efficacy of laundry-associated processes. Table 3 compiles the existing studies as well as summarizes the characteristics of non-enveloped and enveloped viruses with regard to transmission pathways and details to inactivation of viral pathogens associated with textiles; this additional data on the persistence and inactivation of viruses on textiles and laundry-associated material are useful as long as no standard procedure is available to assess the virucidal testing in domestic laundry and can be used competitively to the EN 16777 and EN 14476 [24,25].

Apart from a direct transmission to humans, a transfer of viruses from contaminated textiles to other textiles in a common household laundry process has been described as well [2,49,50,77], presumably during laundering; however, this phenomenon might also take place on dry textile and might especially concern non-enveloped viruses. For instance, Herpes simplex virus (HSV) seems to be more stable in low humidity and at low temperatures [96], so cross-contamination may even occur outside the washing machine, e.g., when handling insufficiently decontaminated laundry. In contrast to that, a complete inactivation of HSV in common household laundry was observed when using a detergent containing activated oxygen bleach [50,60]. Heinzel et al. confirmed a good virucidal efficacy even at 40 °C by conventional household laundry detergents (0.4%) for enveloped viruses like Bovine Viral Diarrhea Virus (BVDV), Vaccinavirus (VACV) as well as for non-enveloped viruses, such as Bovine Parvovirus (BPV), Poliovirus and Simianvirus (SV40); it was shown that the virus particles were not only completely removed from the textiles, but chemically inactivated, leading to a reduction of >5 log [2]. Belonging to the most difficult viruses to inactivate, Noroviruses (NoV) have been shown to be resistant against a great range of chemical agents, thus detergents containing activated oxygen bleach and washing temperatures above 50 °C are required for a complete inactivation when taking the washing machine only into consideration [11,97]. Rotaviruses are inactivated by a common disinfectant ingredients including 70% ethanol, 6% hydrogen peroxide, chlorine, povidone—iodine, hypochlorite (without faecal matter), ultraviolet radiation and heat, not all of them being applicable in laundry. Professional and domestic laundry is mostly followed by a drying cycle at high temperatures (e.g., in a tumble dryer), this means can be considered as well, when estimating the antiviral efficacy of a common laundry process. In some studies, processing steps using heat (80 °C) or even high pressure have also been investigated [98]. Tests on the stability of SARS-CoV on surfaces (polystyrene) showed that SARS-Coronavirus remains infectious for up to six days, in particular in presence of protein load; however, at 56 °C a quick and complete inactivation was observed [16], suggesting the efficacy of laundering and/or drying at high temperatures is sufficient. After a potential transmission of SARS-CoV-2 from contaminated dry surfaces has been discussed [18], MacIntyre et.al. investigated the efficacy of laundering on medical face masks and non-medical face masks (two- layered cotton mask) in a randomised trial [99]. The masks were reused and cleaned on a daily base, either by hand-wash with soap, tap water and air-dried, or by hospital laundry. The study showed that non-medical face masks can be as protective as medical masks, if washed as recommended by WHO (≥60 °C, with detergent) [99,100].

## 5. Conclusions

Although there are not much data available on the antiviral efficacy of laundering, some studies indicate that microorganisms can survive on textiles in a way as to enable them to act as fomites in the transmission of potential pathogens. The role of textiles as fomites in the transmission of healthcare-associated infections has been described previously [66,101,102] and the transfer of different microorganisms of human origin to textiles and vice versa has been investigated before as well [6]. One thing to note, the washing machine has been shown to be a source of re-contamination of textiles [103] and numerous microbial species are transferred to textiles via skin contact by wearing the laundry items, but most of these would be commensal. For example, members of the human skin and mucosal biota can typically be brought to clothes and towels with direct body contact [104]. Although it has been shown before that bacteria and fungi participate in these processes [6], the role of viruses remains unclear. In this regard, however, it has to be considered that viruses, although being able to spread via textiles and laundry processes, are not able to propagate on textiles and hard surfaces and thus must be considered generally different from other pathogens.

Tumble-dryers, which are commonly used at home or in the hospital for drying laundry might be considered for decontamination of virally contaminated textiles as well, either alone or in combination with the above-mentioned laundering processes. Bernard et.al, (2020) found that treatment at 70 °C provide an adequate inactivation of viral pathogens [89] on face masks; this is in line with the proven efficacy of textile decontamination by tumble-drying, which was described previously [105].

Interpretation of data currently suffers from the lack of a uniform procedure for the investigation of antimicrobial effects in laundering processes. While some studies were performed using surrogate viruses of different types, others addressed naturally contaminated textiles. In many cases, the initial microbial load is not reported, and the influence of surrounding parameters has not been described well. Thus, more data on the viral burden of used laundry is needed; moreover, the methods for detecting viruses vary greatly. The use of cell-culture techniques and plaque assays to detect infectious viral particles might not be in line with data obtained with PCR-based techniques. Intact viral RNA appears to remain detectable on surfaces for longer than viruses that retain the ability to infect cells.

Nevertheless, data suggest that laundering can be understood as a reliable and efficient method to decontaminate textiles harboring bacteria and fungi, but also viruses, if a few requirements are met. Inter alia, the use of high temperatures (i.e., 60 °C), activated oxygen bleach containing detergents or chlorine bleach can be assumed to deliver a good antiviral activity, while the use of other ingredients, such as quaternary ammonium compounds may not lead to a sufficient inactivation of all relevant viruses. For enveloped viruses, even 30 °C and a bleach-free detergent must be assumed to deliver a sufficient level of hygiene due to the detergency action on the membrane of enveloped viruses. Thus, laundering is an important means to break the chain of infection also regarding viral pathogens, although hygienic laundering certainly must always be used in line with other hygiene procedures, considering the main routes of infections, most notably the hands.

## Figures and Tables

**Table 1:** Overview of antiviral agents and their activity on different types of virus according to [22,23].

Antiviral Agent/Disinfectant	Non-Enveloped Viruses	Enveloped Viruses
Halogen	+	+
Peroxide	+	+
Aldehyde	+	+
Alcohol	+/−	+
Phenolics	−	+
QAC/Biguanide	−	+
Amine	−	+
Acids	−	+/−
Amphoteric	−	+

(+: active, −: ineffective; +/−: partial active); QAC: Quaternary Ammonium Compound.

**Table 2:** Relevant viruses used as surrogate test viruses [27].

Enveloped Viruses	Non-Enveloped Viruses
Vaccinia Virus/Modified Vaccinia Virus Ankara (MVA)	Adenovirus
Bovine Viral Diarrhea Virus (BVDV)	SV40
Human-Immunodeficiency-Virus (HIV) *	Murine Norovirus
Hepatitis-B-Virus (HBV) *	Bovines Parvovirus/Minute Virus of Mice (MVM)
Hepatitis-C-Virus (HCV) *	Murine Parvovirus (≥30 °C)

* According to Milke et.al., [30]; Reference: EN 16777 [25].

**Table 3:** Overview of studies on the transmission and inactivation of viral pathogens associated with textiles.

Virus	Material/Surface	Main Findings	Methodology/Remarks	Reference
Non-enveloped				
Adenovirus	Cotton test swatches	Proofed virus transfer to sterile laundry 1 log reduction with detergent (linear alkyl benzene sulfonate, sodium carbonate, alkyl sulfate), 4 log with bleach (5.25% sodium hypochlorite), water temperature 20–23 °C More difficult to remove by washing than HAV and rotavirus, temperature resistant up to 56 °C	Inactivation by hypochlorite, cold wash procedures led to small removal rates (log2–3) Surrogate: Hepatitis contagiosa canis (HCC)-Virus	[48,58,62,63,64,65]
Astrovirus	Cotton, paper	Persisted at both 4 °C and 20 °C for two months on dry paper in the presence of faecal material,	Cellulose filter paper as model for nonporous and porous materials	[66]
Hepatitis A/E	Test swatches, cotton fabrics	2 log reduction with detergent (linear alkyl benzene sulfonate, sodium carbonate, alkyl sulfate), 6 log with bleach (5.25% sodium hypochlorite), water temperature 20–23 °C	Cold wash procedures led to small removal rates (1–2 log) Improved reduction with addition of bleach in the wash and rinse cycle (6 log vs. 2 log)	[48]
Mammalian Reovirus	Tap water	Persisted up to 50–60 °C		[67]
MS2 (*Escherichia virus MS2*)	Cotton swatches	Low temperature laundering with 0.2% NOBS and 0.2–1.0% perborate led to 5 log reduction QAC and peracetic acid effective in reduction on surfaces Temperature, ozone, UV, peracetic acid, sodium hypochlorite resistant	Suspension test, temperature stable up to 70 °C and pH stable between 6 and 11 Inactivation between 80–100 C Surrogate for Norovirus and for SARS-CoV-2	[49,68,69,70,71,72]
Norovirus,	Cotton, gauze and diaper material	Survival in the environment for up to 40 days Infectious dose 1–500 particles Complete inactivation after laundering at 60 °C	Surrogates used: murine NoV, feline Caliciviruss (FCV)	[2,11,34,35,58,73,74]
Papillomavirus	Underwear	Persisted on dry inanimate surfaces >7 days	Surrogate: Simianvirus 40	[60,75]
Parvovirus	Cloth, stainless steel	Temperature resistant up to 80 °C,	Surrogate: bovine Parvovirus	[26,76]
Poliovirus	Wool, cotton	Was removed from test swatches when laundering at 40 °C with anionic or non-ionic detergent but lead to cross contamination without inactivation Was reduced by about 99% in cold-water washing conditions without detergent and by 99.98% with cationic or anionic detergents Survived at room temperature for 84–140 days (wool), 42–84 days (cotton) Air drying of fabrics for 20 h resulted in a total reduction of virus of >5 log.	Survival on cotton was greater with saline compared to faecal suspension	[2,48,58,66,77,78]
Rhinovirus A, B, C	Paper handkerchief, nonporous inanimate surfaces	Persisted on dry inanimate surfaces 2 h–7 d.	Paper handkerchief impregnated with iodine or citric acid	[60,79,80]
Rotavirus	Cotton, test swatches, stainless steel, plastic, cloth	2 log reduction with detergent (linear alkyl benzene sulfonate, sodium carbonate, alkyl sulfate), 5 log with bleach (5.25% sodium hypochlorite), water temperature 20–23 °C 3–4 log at high relative humidity (RH) Cold wash procedures led removal rates of 1–2 log Survival is better on cloth than on paper Persisted in freshwater for several days Can survive and stay infective for several months in faecal material at 10 °C and on contaminated surfaces Stable to pH extremes	Improved reduction with addition of bleach (5 log vs. 2 log reduction)	[21,48,66,81,82,83]
Simianvirus 40	Cloth, stainless steel	persisted up to 50–60 °C		[26,67,84]
Enveloped				
Alphacoronavirus 1	stainless steel	Survived at 4 °C for >28 d Inactivation more rapidly at 40 °C than at 20 °C, slowest inactivation at low RH. >3.5 log when treated with ethanol (71%) >4.9 log reduction when treated with H_2_O_2_	Surrogate for SARS-CoV [8] Previous known as/member viruses: Transmissible gastroenteritis coronavirus (TGEV), feline/canine CoV	[8,59,85]
Bovine Viral Diarrhea Virus	Cotton	Washing led to removal rates of >4 log at 20 °C for detergent (0.4%) and for ethanol (50%) >4.5 log Transfer of viruses to the washing liquor or not contaminated textiles	Suspension test in hard water, low and high protein loads, different temperatures, testing times of 5–60 min BVDV as surrogate for Hepatitis C virus (HCV)	[2,24,74]
HSV-1 (herpes simplex virus 1), (HSV-2)	Plastic, cotton fabrics	Persisted on cloth for less than 2 h Resisted laundering process at 40 °C Adhering particles 48 hours after domestic laundry	Surrogate: Suid herpesvirus 1 (SuHV-1) stable up to 50 °C	[50,65,75,86]
Human Coronavirus (HCoV) 229E; OC43; NL63	Polystyrene, paper, disposable gowns, cotton gowns	Remained infectious on inanimate surfaces at room temperature for up to 9 days. Ethanol (62–71%): 2–4 log Sodium hypochlorite (0.1–0.5%): >3 log Glutardialdehyde (2%): >3 log Benzalkonium chloride (0.04%), Sodium hypochlorite (0.06%) and Ortho-phtalaldehyde (0.55%) were less effective	1 min exposure time Viable virus not detected after drying; viral RNA detectable for up to 7 days [87]	[10,16,60,88]
Influenza virus (A + B)	Cotton, handkerchief, paper, stainless steel	3–5.5 log moist-air heating Infectious dose:1–10 virus particles Survival for 8–12 h on fabrics, but up to 24–48 h on hard surfaces Measurable quantities of virus were transferred from tissues to hands at 15 min. Survived longer on plastic and stainless steel (24–48 h) vs. paper and tissue (6–8 h)	Moist heating treatment also allowed inactivation compared to dry air heating	[12,89]
Middle East respiratory syndrome-related coronavirus (MERS-CoV)	Steel, plastic	Viable virus detected after 48 h at 20 °C/40% RH. Less survival at 30 °C/80% RH (8 h) and 30 °C/30% RH (24 h). Half-life ranged from 0.5 to 1 h.	Together with CoV-2 great capacity to survive on dry surfaces compared to other human coronaviruses (229E, OC43, and NL63).	[18,90]
Parainfluenza virus (PIV)	No absorptive/absorptive surface	Survived up to 4 h Could be isolated from table tops, chair arms and desks in office buildings in 5 US cities		[91,92]
Respiratory syncytial virus (RSV)	Cloth	Contaminated surfaces remained infectious for up to 6 h (countertops) Was recovered from cloth gowns and paper tissue (30–45 min) and from skin for up to 20 min		[6]
SARS-CoV-1	Plastic, stainless steel	Positive tested surfaces in domestic application (swab) Remained infectious for 14 days at 4 °C, and for 2 days at 20 °C Compared to CoV-2 shorter half-life on cardboard	pH sensitive	[8,9,93,94,95]
SARS-CoV-2	Surgical masks/FFP2 respirators, cloth; Cotton swatches, stainless steel (MHV, M-CoV)	Recovered from surface of surgical masks after 7 days Inactivated by moist-air heating (75% RH) at 70 °C for 1 h Extremely stable over a wide pH range (pH 3–10)	Mask material: Nanofibers made with polypropylene Surrogate viruses for CoV-2: Mouse Hepatitis Virus, (MHV); murine coronavirus (M-CoV)—3.9 log reduction with 70% ethanol, Inactivation more rapidly at 40 °C than at 20 °C, slowest inactivation at low RH	[19,89]
Vaccinia virus	Wool, cotton	>4 log with laundry detergent 0.4%, 20 °C/5 min, in standards suspension tests		[2,26,67,78]

## Data Availability

Not applicable.
